# The Multifaceted Interplay between Atrial Fibrillation and Myocardial Infarction: A Review

**DOI:** 10.3390/jcm10020198

**Published:** 2021-01-07

**Authors:** Alban Belkouche, Hermann Yao, Alain Putot, Frédéric Chagué, Luc Rochette, Nicolas Danchin, Laurent Fauchier, Marianne Zeller, Yves Cottin

**Affiliations:** 1 Department of Cardiology, University Teaching Hospital of Dijon Bourgogne, 21000 Dijon, France; alban.belhouche@chu-dijon.fr (A.B.); hermannyao@gmail.com (H.Y.); frederic.chague@chu-dijon.fr (F.C.); 2 Department of Geriatry, University Teaching Hospital Dijon Bourgogne, 21000 Dijon, France; alain.putot@chu-dijon.fr; 3 Team PEC2, EA 7460, University of Burgundy, 21078 Dijon, France; luc.rochette@u-bourgogne.fr (L.R.); marianne.zeller@u-bourgogne.fr (M.Z.); 4 Department of Cardiology, University Teaching Hospital of Georges Pompidou, 75015 Paris, France; nicolas.danchin@aphp.fr; 5 Department of Cardiology, University Teaching Hospital of Trousseau and University François Rabelais, 37000 Tours, France; laurent-fauchier@univ-tours.fr

**Keywords:** atrial fibrillation, chronic coronary syndrome, bleeding, type 1 and type 2 myocardial infarction

## Abstract

This review was conducted to emphasize the complex interplay between atrial fibrillation (AF) and myocardial infraction (MI). In type 1 (T1) MI, AF is frequent and associated with excess mortality. Moreover, AF after hospital discharge for T1MI is not rare, suggesting the need to improve AF screening and to develop therapeutic strategies for AF recurrence. Additionally, AF is a common trigger for type 2 MI (T2MI), and recent data have shown that tachyarrhythmia or bradyarrhythmia could be a causal factor in, respectively, 13–47% or 2–7% of T2MI. In addition, AF is involved in T2MI pathogenesis as a result of severe anemia related to anticoagulants. AF is also an underestimated and frequent cause of coronary artery embolism (CE), as a situation at risk of myocardial infarction with non-obstructive coronary arteries. AF-causing CE is difficult to diagnose and requires specific management. Moreover, patients with both AF and chronic coronary syndromes represent a therapeutic challenge because the treatment of AF include anticoagulation, depending on the embolic risk, and ischemic heart disease management paradoxically includes antiplatelet therapy.

## 1. Introduction

Acute or chronic coronary syndrome (CCS) and atrial fibrillation (AF) are common conditions with mutual interplay. Patients with AF often suffer from CCS, with a prevalence ranging from 36 to 86%, and there is an increased risk of AF in patients with acute myocardial infarction (MI) [1].

**Figure 1:**
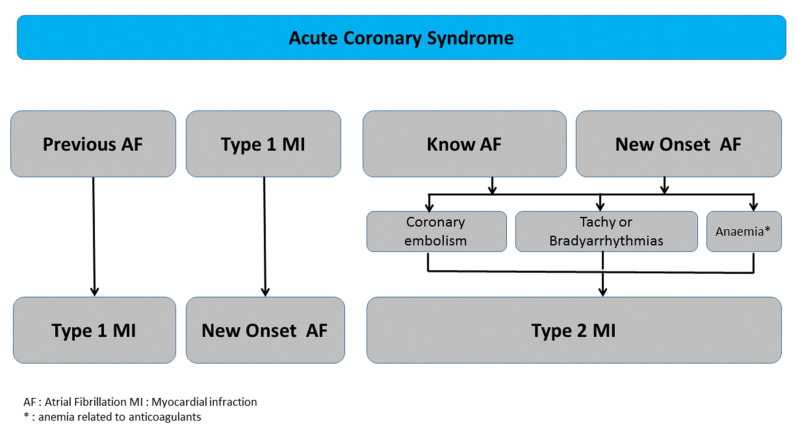
Multifaceted interplay between atrial fibrillation and myocardial infarction.

Elevated cardiac troponin (cTn) levels indicate myocardial cell damage/death but do not reflect the underlying etiology. The current universal definition of MI proposes a classification for MI subgroups including: type 1 MI (T1MI) and type 2 MI (T2MI) [2,3]. The classification of T1MI and T2MI has varied widely among studies and a standardized approach with clear definitions is needed to avoid misclassification and ensure appropriate patient care. T1MI is due to plaque rupture with thrombus, while T2MI is a result of myocardial supply/demand mismatch. Thus, two sub-types of relationships may occur between AF and MI: (a) in T1MI, a number of patients have a history of AF or experience AF as a complication, and (b) in T2MI, not only AF per se, whether tachyarrhythmias or bradyarrhythmias, but also AF treatments such as drug-induced bradycardia or anticoagulant-related anemia are major causes of myocardial oxygen mismatch (Figure 1). The objective of this review was to emphasize the complex interplay between AF and MI, supported by most recent findings from the literature, including frequency and prognosis.

## 2. Eligibility Criteria and Data Extraction

All observational studies that analyzed presenting symptoms, baseline characteristics, interventions, and mortality outcomes between atrial fibrillation and myocardial infraction, published after 1 January 2010, were identified by a search conducted in the databases of PubMed, EMBASE, Cochrane Central Register of Controlled Trials (CENTRAL) and MEDLINE using the MeSH terms “atrial fibrillation”, “myocardial infarction”, “type-1 myocardial infarction”, “type-2 myocardial infarction”, and “coronary embolism”. Studies were included if they met the following criteria: (1) included a comparison of AF versus non-AF in patients with MI and (2) had data for outcome variables of interest.

### 2.1. AF and T1MI

Many studies have shown a high prevalence of CCS (30–40%) in AF patients based on medical history, angina symptoms, and ECG abnormalities [4]. In patients with non-valvular AF, data from randomized controlled trials (RCTs) comparing warfarin and oral anticoagulants (OACs) showed similar results [5]. AF was associated with a history of MI in 14–18% of patients, and the follow-up of patients taking OACs or warfarin showed an incidence of MI per year ranging from 0.51 to 1.1% [5]. Overall, a lower rate of MI has been reported in RCT than in registries (0.4–1.3%/year) [6].

A history of permanent or paroxysmal AF is common in acute MI. Meta-analyses have consistently reported an incidence of prior AF ranging from 2 to 13% [7,8,9,10,11,12,13,14,15,16] (Table 1). The largest cohort (155,071 patients) found an incidence of 7.9%, including 4.9% of patients with a history of paroxysmal AF (defined by sinus rhythm (SR) on admission and discharge ECG) and 3% with chronic AF (defined by AF on both admission and discharge ECG) [16]. After adjustment, prior AF was associated with in-hospital major bleeding but was not associated with in-hospital adverse outcomes such as death, re-infarction, or heart failure [16] (Table 1).

In contrast, patients with prior AF and MI had poorer long-term prognoses than patients with SR. A meta-analysis from 2011 found an increased long-term mortality risk in patients with prior AF [7]. In addition, MI patients with prior AF had a higher risk of cardiovascular (CV) events (all-cause mortality, recurrent MI, or ischemic stroke), with an event rate/100 persons of 84.0 (prior paroxysmal AF), 115 (prior persistent/permanent AF), and 45.2 (SR) [7]. When compared with SR patients, the adjusted HR was found to be 1.26 (95%CI (1.1–1.4)) for prior paroxysmal AF and 1.31 (95% CI (1.1–1.5)) for chronic prior FA [7].

The pathophysiology of acute MI and reperfusion, notably the temporal and spatial evolution of ischemic and reperfusion injury, is multifactorial in relationship with the different modes of cell death and the resulting coronary microvascular dysfunction. The remodeling of the left atrium includes a series of morphological and functional changes that occur as an adaptive response to factors that lead to AF, and MI is one of the major pathophysiological conditions that promote the onset of AF [17]. Between 2 and 25% of patients without prior AF will develop it during or after acute MI [7,8,9,10,11,12,13,14,15,16] (Table 1). In addition, new-onset AF (NOAF) is an independent, long-term risk predictor of stroke, major adverse cardiac and cerebrovascular events, and all-cause mortality, even if normal rhythm is restored prior to discharge [7,8,9,10,11,12,13,14,15,16,17,18].

This was shown in Lau’s study, which included 3393 patients (755 ST segment elevation MI (STEMI), 1942 with high-risk non-ST segment elevation MI and 696 with intermediate risk non-ST segment elevation MI) [12]. While 387 patients (11.4%) had history of AF before ACS, 149 patients (4.4%) had their first episode of AF during acute MI. Compared to patients without an episode of AF, patients with NOAF had an increase in mortality (HR 2.2, 95% CI (1.0–4.6), *p* = 0.04) and an increase in major bleeding (HR 5.8, 95% CI (3.1–10.6), *p* < 0.001) at one year of follow-up after adjustment.

There is currently some controversy as to whether the appearance of NOAF during acute MI is an indication for long-term anticoagulation therapy. To our knowledge, no RCT on the indication of anticoagulation in the event of new onset AF has been published. Moreover, these data must be considered along with the concept of “reversible AF” and the impact of early-AF or late-AF in the acute phase.

Asanin et al. followed acute MI patients with NOAF but sinus rhythm at hospital discharge for a seven-year period [19]. Patients with NOAF had more frequent AF than those without NOAF (10.4% vs. 2.7%, respectively; *p* < 0.0001), and AF recurrence during follow-up and no anticoagulation at discharge were independent predictors of stroke. A cut-off duration of 3.5 h-long AF within the first 48 hours of acute MI had the highest sensitivity for the prediction of low long-term risk of stroke obtained by receiver operating characteristic (ROC) curve analysis. Among patients who did not receive anticoagulants at discharge, the patients with short-duration AF (<3.5 h) were less likely to experience stroke and AF recurrence during follow-up, while those in the other group were more likely to develop these complications (10.8%, *p* = 0.038 and 13.5%, *p* = 0.019, respectively) [19].

In 2020, Axelrod et al. published a study that included 7061 patients with NOAF during acute MI, excluding patients with pre-existing AF and those who underwent coronary artery bypass grafting (CABG) surgery or who died during hospitalization [20]. The cohort was divided into three groups: 1: acute MI without AF; 2: AF onset and resolution within the first 24 h of admission and no further AF events during the index hospitalization (1.4%); and 3: late AF either starting or continuing beyond the first 24 h of admission (2.5%). With a median follow-up of six years, stroke rates were only higher in the late-AF group versus the no-AF group but not in the early-AF group (10.6% vs. 4.2% and 5.3%, respectively) (*p* < 0.001). In addition, unadjusted death rates were higher in the late-AF and early-AF groups versus the no-AF group (55.3%, 43.2%, and 29.2%, respectively) (*p* < 0.01).

### 2.2. Silent AF

Silent or subclinical asymptomatic atrial fibrillation (SAF) has gained extensive interest in the epidemiologic, neurologic, and cardiovascular areas. The electrophysiological and mechanical effects of symptomatic and SAF are equivalent. Therefore, SAF should be considered when estimating the prevalence of the disease and its impact on morbidity, mortality, and quality of life.

New-onset symptomatic AF is common within the first days after MI, but silent AF is three-times more frequent [21]. Using continuous ECG monitoring (CEM) ≥ 48 h after admission for MI, Stamboul et al. demonstrated that 135/849 patients (16%) developed silent AF while only 45/849 patients developed symptomatic AF (5%) [21]. Compared to patients without AF, patients with silent AF were significantly older, more frequently women, and less likely to be smokers. In addition, like symptomatic AF, silent AF is associated with a poor prognosis at one year post-ACS, with a significant increase in CV mortality and hospitalizations for heart failure compared to patients without AF, respectively, at 5.7% vs. 2.0% (*p* < 0.001) and 6.6% vs. 1.3% (*p* < 0.001). Transient NOAF was considered by Wi et al. as AF occurring within acute MI without a prior history and not documented for one month after discharge (4.8% of patients). Transient NOAF was associated with the poorest clinical outcomes and was a significant independent predictor of major adverse cardiac events (MACEs) and death in acute MI patients at one month, two years, and five years [22].

### 2.3. AF in Post MI

An acute coronary occlusion results in marked electrophysiological alterations that can induce ventricular tachycardia or ventricular fibrillation, often heralding sudden cardiac death. However, it is important to remember that during the infarct-healing stage, hemodynamic and structural changes can lead to left ventricular dilatation and dysfunction, whereas the accompanying fibrosis forms the substrate for re-entrant circuits sustaining frequent dysrhythmias such as AF. The incidence of AF in patients with a history of acute MI is higher than in the general population, with a cumulative incidence of 6–21% at five years after acute MI compared with an incidence of only 3% in the general population [23,24,25]. AF episodes after acute MI generally occur within the first year [14].

The Rochester Epidemiology Project (3220 patients from 1983 to 2007) was the first important registry to assessed AF occurrence after MI [14]. AF was identified before MI in 304 patients, while 729 developed AF after MI (218 (30%) within 2 days, 119 (16%) between 3 and 30 days, and 392 (54%) > 30 days post-MI. The cumulative incidence of AF after MI at five years was 19% and did not change over the calendar year of MI (the incidence of AF was the same regardless of when the MI occurred). During a mean follow-up of 6.6 years, 1638 deaths occurred. AF was associated with an increased risk of death independently of clinical characteristics at the time of MI and heart failure. This risk was the greatest for AF occurring >30 days post MI (HR 1.63 (1.37–1.93) for AF within 2 days, HR 1.81 (0.45–2.27) for AF between 3 and 30 days, and 2.58 (2.21–3.00) for AF > 30 days post MI) [24].

A meta-analysis by Jabre et al. that was published in 2011 and included 43 studies and 278,854 patients demonstrated an increased risk of mortality associated with the presence of AF in the stetting of MI [7]. AF was associated with an increased mortality risk of at least 40% compared with sinus rhythm (HR 1.46 (1.35–1.58)). In addition, the poor prognosis persisted regardless of the timing of AF, and the odds ratio of mortality for new AF with no prior history of AF was 1.37 ((1.26–1.49); I(2) = 28% (nine studies)), and the odds ratio for prior AF was 1.28 ((1.16–1.40); I(2) = 24% (four studies)). The sensitivity analysis for new AF studies adjusting for confounding factors did not show a decrease in the risk of death [7].

In conclusion, the risk of AF after hospital discharge is not an epiphenomenon, which suggests a need to develop monitoring and therapeutic strategies for screening AF recurrence after acute MI.

### 2.4. AF and T2MI

As we previously reported, T2MI is defined as MI whereby a condition other than atherosclerotic coronary artery disease creates an imbalance between myocardial oxygen supply and demand. Observational studies have shown that compared with patients with T1MI, patients with T2MI were older and more often female, had more hypertension, and had diabetes mellitus.

Though the studies described in the previous chapters are relevant, they preceded the universal definition of MI established in 2018. It is therefore important to highlight that an unknown number of patients presenting T2MI were thus included in the analyses. To our knowledge, no study has specifically addressed AF as a trigger for T2MI. T2MI may occur with or without obstructive coronary artery disease, and the threshold for T2MI is lower in patients with fixed obstructive CCS [5,13]. A modest revision for classifying myocardial infarction T2MI, splitting it into type 2A with and type 2B without fixed obstructive CCS, has recently been proposed [25]. AF can lead to a T2MI through either increased oxygen demand due to tachyarrhythmia or reduced supply via bradyarrhythmia related or not to drugs with bradycardic effects or anemia related to anticoagulants [3] (Figure 1).

Among studies using the 2007 and 2012 universal definition of MI, the prevalence of T2MI in patients with acute MI ranged from 2% to 58% [3]. Wide variations in the prevalence of T2MI were also observed between sites in the same study (0–13%) [25,26,27,28,29,30,31,32,33,34,35,36,37,38,39,40,41,42,43,44] (Table 2, Table 3 and Table 4). This variation was likely influenced by many factors including patient populations, sensitivity, and diagnostic thresholds of cTn assays, rate and types of additional cardiac investigations, limitations of diagnostic criteria, and the interpretation of these criteria by adjudicators of MI subtypes. In most studies, both short- and long-term mortality were higher among patients with T2MI than in patients with T1MI, and differences in T2MI mortality between studies are likely explained by patient selection [25,26,27,28,29,30,31,32,33,34,35,36,37,38,39,40,41,42,43,44]. Two recent meta-analyses provided major information on this subject. First, in 2016, Gupta et al. included 25,872 patients, of whom 2683 (10%) had T2MI [45]. Compared to T1MI, patients with T2MI had significantly higher in-hospital, 30-day, and 1-year mortality rates, respectively, of 4.7% vs. 15%, 5.3% vs. 17.6%, and 13% vs. 27%. In addition, the most common triggers of T2MI were stress (20%), sepsis (19%), arrhythmia (19%), heart failure (15%), and anemia (12%) [45,46]. 

#### 2.4.1. AF as a Trigger for T2MI

There are diverse underlying mechanisms that can cause T2MI [26,27,28,29,30,31,32,33,34,35,36,37,38,39,40,41,42,43,44] (Table 2, Table 3 and Table 4), and the co-existence of multiple triggers is frequent (24–33% of cases). AF can induce T2MI either through an increase in myocardial oxygen demand (as in the case of tachyarrhythmia), through reduced supply (in the case of bradyarrhythmia) or as a result of severe anemia related to anticoagulants. Moreover, in patients with tachyarrhythmia, an increased heart rate reduces ventricular filling time, which results in a decreased coronary artery perfusion during diastole. Table 2, Table 3 and Table 4 summarize the various triggers for T2MI and indicate that tachyarrhythmia could occur in 13–47% of T2MI cases. Anticoagulant treatment is the leading cause of bleeding, and the rate of major bleeding established by the International Society on Thrombosis and Hemostasis (ISTH) ranges from 2.3% to 4.5% per year [47]. The prescription rate for anticoagulants ranges from 4 to 15% for T1MI and from 9.7–27% for T2MI (Table 2 and Table 3) [32,35,36,37,38]. The two studies that addressed anemia as a trigger for T2MI found this condition in 7% and 13% of cases (Table 2 and Table 3) [32,38].

Prospective or retrospective studies are thus urgently needed to address this important issue, both in the acute phase of MI and in the long term.

#### 2.4.2. AF-Related Risk Factors and T2MI Prognosis

Due to clearly identified risk factors (i.e., anemia related to anticoagulation), AF therefore associated with non-cardiovascular excess mortality in the long term after MI. Further studies are urgently needed to identify the impact of underlying heart disease, heart rhythm disorders, and/or therapeutics on T1MI or T2MI, as well as to determine cut-off values for hemoglobin and heart rate.

Only two articles have provided information on the prognostic impact of the T2MI trigger. In a recent study including 5460 MI patients, 1054 were classified as T2MI. The age- and sex-adjusted rates of all-cause mortality at five years were significantly higher after T2MI versus after T1MI (52% versus 31%, respectively, *p* < 0.001) [48]. In addition, after adjustment for age, sex, and baseline comorbidities, T2MI remained an independent predictor of all-cause mortality but not CV mortality. Over five years of follow-up, rates of all-cause mortality with arrhythmia, anemia, or hypoxia as trigger were, respectively, 52%, 70%, and 80%. Only one study provided a prognostic score (Troponin Assessment for Risk stRatification of patients without Acute COronary atherothrombosis (TARRACO) risk score) for major adverse CV events at 180 days in patients with T2MI or non ischemic myocardial injury (NIMI) [49]. The score included six variables: (1) age, (2) hypertension, (3) lack of chest pain, (4) dyspnea, (5) anemia, and (6) troponin > 5 upper reference limit (URL). Patients were classified into low-risk (score 0–6) and high-risk (score ≥ 7) categories. Rates of major adverse CV events were five times higher in high-risk patients compared to low-risk patients, respectively, at 78.9 vs. 15.4 events/100 patient-years. Anemia, whether linked to anticoagulants or not, is therefore a major prognostic factor after T2MI. In addition, in the 611 patients of the TARRACO derivation cohort (median age of 78 years; 55% men), the authors reported that 33.5% had AF. Though this study did not provide any information on anemia related to anticoagulants for AF, there was an increased risk of bleeding in AF patients receiving an anticoagulant in combination with one-to-two antiplatelet agents. RCTs have shown that patients receiving triple antithrombotic therapy (TAT) or dual antiplatelet therapy (DAPT) have clinically significant bleeding rates ranging from 9 to 26%, depending on the dosage, molecule, and treatment duration.

#### 2.4.3. T2MI and CAD

As we previously reported, when compared with patients with MT1I, patients with T2MI are older, more frequently female, and more likely to have chronic kidney disease and comorbidities. Patients with T2MI are also less likely to have significant coronary artery disease (CAD) and to undergo coronary angiography, percutaneous coronary intervention, and CABG surgery.

The prognostic significance of underlying CAD during the management of T2MI is a key issue. In a study by Chapman et al., the authors demonstrated that the presence of CAD is an independent predictor of MACE at five years in patients who presented with T2MI [36]. To our knowledge, there are no specific data regarding the efficacy of validated therapies to prevent T2MI. In the latest universal definition of MI in 2018, the authors advise managing the triggering factor first in an acute setting. If this is demonstrated, routine screening for CAD after T2MI would be justified, especially in the case of an FA trigger.

### 2.5. AF Patients and Coronary Embolism

A coronary artery embolism (CE) is recognized as a major cause of MINOCA (myocardial infarction with non-obstructive coronary arteries). Though AF is one of the most frequent causes of ischemic stroke, MI related to CE is rare. The prevalence, clinical features, and prognosis of CE remain insufficiently characterized. Among 1776 consecutive patients who presented with de novo acute MI, CE was diagnosed based on histological, angiographic, and other criteria linked to diagnostic imaging. The prevalence of CE was found to be 2.9% (*n* = 52), including eight (15%) patients with multi-vessel CE. AF was by far the most common cause (*n* = 38, 73%). Only 39% of patients with CE were treated with vitamin-K antagonists, and the median international normalized ratio (INR) was 1.42 (range of 0.95–1.80) [50]. During a median follow-up of 49 months, five AF patients had relapses for CE and thromboembolism [50]. CE is an under-recognized etiology of MI, and it is associated with significant morbidity and mortality. Cardiac magnetic resonance imaging (MRI) can identify ischemic myocardial sequelae when there is a suspicion of CE, in particular without obstructive CCS. 

## 3. Conclusions

AF is a frequent complication that is associated with an increased mortality in patients with all types of MI. However, only few studies have specifically addressed the acute management of AF with invasive and/or pharmacological strategies. AF is also an underestimated and frequent cause of coronary artery embolism (CE) as a situation at risk of myocardial infarction with non-obstructive coronary arteries. Moreover, AF is difficult to diagnose and requires specific management. In addition, patients with AF and CCS represent a particular therapeutic challenge because of the diverse antithrombotic therapies: the treatment of AF should include anticoagulation, but depending on the embolic risk, antiplatelet therapy may also be considered for ischemic heart disease management. Further studies are necessary to further clarify the risks and benefits of oral anticoagulants in CAD without aspirin medication.

## Figures and Tables

**Table 1:** Major studies published regarding the acute coronary syndrome-atrial fibrillation relationship: major finding [7,8,9,10,11,12,13,14,15,16].

Authors	Nb	Atrial Fibrillation (AF) Incidence			Follow-Up	Event
		Any AF	Prior AF	NOAF		
Jabre et al.; Meta-analysis [7] - AF vs. sinus rhythm - AF before MI vs. sinus rhythm - New Onset AF vs. sinus rhythm	43 studies 278,854 patients	4–25%	2–13%	6–17%	Hospitalization to 8 years	Mortality 1.46 (1.35–1.58) 1.27 (1.16–1.40) 1.37 (1.26–1.49)
He et al.; Meta-analysis [8]	11 studies 9570 patients			4.8–20.7%		
Luo et al.; Meta-analysis [9] - AF vs. sinus rhythm	14 studies 292,774 patients			4.5–13.7%	Hospitalization to 3 years	Ischemic stroke 2.84 (1.91–4.23)
Kundu et al.; Registry [10] - New onset AF vs. sinus rhythm	Registry 6384 patients		7.7%	10.8%		In-hospital death 19.6% vs. 7.9% 2.3 (1.9–2.9) In hospital stroke 3.6% vs. 1.4% 2.5 (1.6–4.1) 30 day post-discharge death 8.3% vs. 5.1% 1.5 (1.2–1.8)
Guenancia et al. [11]	Registry 1621 patients	25%	11%	17% - Silent AF: 13% - Symptomatic AF: 4%		1 year mortality 21% vs. 7% 1 year stroke 0.9% vs. 1%
Lau et al. [12]	3393 SCA NSTEMI	15.8%	4.4%	11.4%	1 year mortality New onset vs. None AF: 1.36 (0.84–2.20) Previous AF vs. no AF: 1.42 (1.01–1.99) 1 year MI New onset vs. None AF: 1.80 (1.13–2.86) Previous AF vs. no AF: 0.82 (0.54–1.24) 1 year stroke Previous AF vs. no AF: 1.01 (0.21–4.78) 1 year MCE New onset vs. None AF: 1.66 (1.18–2.33) Previous AF vs. no AF: 1.13 (0.86–1.49)	
Congo et al. [13] - New Onset AF vs. sinus rhythm	6957 patients	14%	8%	5%	STEMI	In hospital mortality 13.4% vs. 3.8% 1.19 (0.62–2.27)
Jabre et al. [14]	3220 patients	32.1%	8.5%	Early AF ≤ 2 days: 6.8% Intermediate AF 2 < AF ≤ 30 days: 3.7% Late AF > 30 days: 12.1%		
Hofer et al. [15]	1372 patients	17.5%	6.6%	10.9%		Long term cardiovascular mortality Do novo AF vs. no AF 1.45 (1.19–2.57) Preexisting AF vs. no AF 0.70 (0.35–0.98)
Pokorney et al. [16]	155,071 patients	15.5%	7.9% - History of Paroxysmal AF (sinus rhythm on arrival and discharge ECG): 4.9% -Persistent/Permanent AF (AF on both arrival and discharge ECG): 3.0%	7.6% - New Onset (AF on arrival ECG but sinus rhythm on discharge ECG): 3.7% - New Onset (AF on discharge ECG): 3.9%	CV at 90 days (all-cause mortality, MI or ischemic stroke): - New Onset (AF on arrival ECG but sinus rhythm on discharge ECG): 1.23 (1.1–1.4) - New Onset (AF on discharge ECG): 1.36 (1.2–1.6) - History of Paroxysmal AF (sinus rhythm on arrival and discharge ECG): 1.36 (1.1–1.4) - Persistentt/Permanent AF (AF on both arrival and discharge ECG): 1.31 (1.1–1.5) All AF: 1.28 (1.2–1.4)	

**Table 2:** Major studies published regarding the type 1 MI/type 2 MI (T1MI/T2MI) regarding known AF, known chronic coronary syndrome (CCS), and triggers of T2MI [25,26,27,28,29,30,31]. MACE: major adverse cardiac event.

Authors		N (%)	Age (Years) T2	Known CCS before MI (%)	Known AF before MI	Anticoagulant at Admission	Triggers for Type 2 MI	Outcomes
Landes et al. [26]	T1MI	107 pts	72 ± 12.5	46.7%	ND	ND		All-cause mortality T2MI vs. matched T1MI (HRadj-95% CI) 30 days 7.17(131–38.9) *p* = 0.023 1 year 3.42 (1.51–7.75) *p* = 0.003 5 years 2.08 (1.14–3.81) *p* = 0.17 MACE T2MI vs. matched T1MI (HRadj-95% CI) 30 days 1.07(0.49–2.31) *p* = 0.871 1 year 1.05 (0.51–2.17) *p* = 0.895 5 years 1.02 (0.59–1.76) *p* = 0.950
	T2MI	107 pts matched *	74 ± 10.4	63.6%	ND	ND	1. Sepsis 36% 2. Tachyarrhythmia AF: 30% 3. Post-surgically: 25% Bradyarrhythmia: ND Multiple provoking conditions: ND	
Smilowitz et al. [27]	T1MI	137 pts	70.9 ± 12.7	19.0% (MI)%	ND	ND		In-hospital mortality T1MI vs. T2MI Cardiovascular death: 5.8% vs. 2.1% *p* = 0.18 Non-cardiovascular death: 7.3% vs. 9.6% *p* = ND In-hospital or 30-day mortality T1MI vs. T2MI Cardiovascular death: 6.6% vs. 4.1% *p* = 0.45 Non-cardiovascular death: 8.0% vs. 10.3% *p* = ND
	T2MI	146 pts	74.3 ± 12.2	19.2% (MI)	ND	ND	1. Tachyarrhythmia: 29.5% 2. Severe Hypertension: 19.9% 3. Severe Anaemia 19.9% Bradyarrhythmia: 6.8% Multiple provoking conditions: 42.5%	
Sandoval et al. [28]	T1MI	77 pts	61 ± 14	31%	9%	ND		180 day all-cause mortality T1MI vs. T2MI 8% vs. 13% *p* = NS 180 day post-discharge MACE T1MI vs. T2MI 10% vs. 16% *p* = NS
	T2MI	140 pts	60 ± 15	17%	11%	ND	1. Tachyarrhythmia: 47.1% 2. Severe Hypertension: 42.1% 3. Respiratory Failure: 40.7% Bradyarrhythmia: 3.6% Multiple provoking conditions: ND	
Smilowitz et al. [29]	T1MI					ND		In hospital death: 6%
	T2MI	255	76 ± 13	50%	38%	ND	1. Surgery: 35% 2. Anemia: 32% 3. Sepsis: 31% Bradyarrhythmia: 2% Multiple provoking conditions: 8%	
Sabby et al. [30]	T1MI	397 pts	71 ± 14	24% (MI)	12.6%	ND		ND
	T2MI	144 pts	75 ± 15	27.1% (MI)	23.6%	ND	1. Anemia: 21% 2. Respiratory Failure: 21% 3. Tachyarrhythmia: 19% Bradyarrhythmia: 3% Multiple provoking conditions: 10%	
Stein et al. [31]	T1MI	2691 pts	64 ± 13	28.1% (MI)	ND	ND		1-year mortality T1MI vs. T2MI 8.6% vs. 23.9% *p* < 0.0001
	T2MI	127 pts	75 ± 12	44.4% (MI)	ND	ND	1. Anaemia: 31% 2. Sepsis: 24% 3. Tachyarrhythmia: 17% Bradyarrhythmia: ND Multiple provoking conditions: 24%	

*: matching for sex, ST segment elevation.

**Table 3:** Major studies published regarding the T1MI/T2MI regarding known AF, known CCS, and triggers of T2MI [32,33,34,35,36,37,38].

Authors		N (%)	Age (Years) T2	Known CCS before MI (%)	Known AF before MI	Anticoagulant at Admission	Triggers for Type 2 MI	Outcomes
Baron et al. [32]	T1MI	17,488 pts	71 ± 12	30.4% (MI)	ND	5.3%		1-year mortality T1MI vs. T2MI 13.5% vs. 23.7% *p* < 0.001
	T2MI	1403 pts	76 ± 11	40.1% (MI)	ND	10.2%	1. Tachyarrhythmia: 24% 2. Respiratory Failure: 19% 3. Sepsis: 18% 4. Anemia: 13% Bradyarrhythmia: ND Multiple provoking conditions: ND	
Lambrecht et al. [33]	T1MI	360 pts	70 ± 13	ND	8.9%	ND		All-cause mortality at 3 years T1MI vs. T2MI 31.7% vs. 62.2% *p* < 0.0001
	T2MI	119 pts	75 ± 11	ND	21.0%	ND	ND	
Cediel et al. [34]	T1MI	376 pts	66	ND	31.9%	ND		2-year mortality T1MI vs. T2MI 19.7% vs. 39.7% *p* < 0.001
	T2MI	194 pts	79	ND	21.1%	ND	1. Heart failure: 28% 2. Tachyarrhythmia: 19% 3. Respiratory Failure: 19% Anaemia: 0% Bradyarrhythmia: 0% Multiple provoking conditions: ND	
Shah et al. [35]	T1MI	1171 pts	68 ± 14	45%	ND	4%		1-year mortality T1MI vs. T2MI 16% vs. 37% *p* < 0.001
	T2MI	429 pts	75 ± 13	45%	ND	10%	ND	
Sarkisian et al. [25]	T1MI	2673 pts	ND	ND	ND	ND		ND
	T2MI	1089 pts	71 ± 14	17% (MI)	29%	ND	1. Sepsis: 30% 2. Heart Failure: 16% 3. AF: 10% Anaemia: ND Bradyarrhythmia: 7% Multiple provoking conditions: 33%	
Chapman et al. [36]	T1MI	1171 pts	68 ± 14	45%	ND	4.5%		5-year mortality T1MI vs. T2MI 31.0% vs. 56.1% *p* < 0.001
	T2MI	429 pts	75 ± 14	45%	ND	9.7%	1. Arrhythmia: 19% 2. Pneumonia: 13% 3. Heart Failure: 12% Anaemia: ND Bradyarrhythmia: ND Multiple provoking conditions: ND	
Putot et al. [37]	T1MI	2036 pts	67 ± ND	20%	7.9%	7.9%		In-hospital mortality T1MI vs. T2MI 6.1% vs. 14%, *p* < 0.001
	T2MI	947 pts	81 ± ND	31%	23.7%	23.7%	1. Acute infection: 36% 2. Heart Failure: 34% 3. Anemia: 7.8% Bradyarrhythmia: ND Multiple provoking conditions: ND	
Putot et al. [38]	T1MI	365 pts	72 ± ND	30%	ND	15%		In-hospital mortality T1MI vs. T2MI 7% vs. 15%, *p* < 0.001
	T2MI	254 pts	82 ± ND	15%	ND	27%	ND	

**Table 4:** Major studies published regarding the T1MI/T2MI regarding known AF, known CCS, and triggers of T2MI [39,40,41,42,43,44].

Authors		N (%)	Age (Years) T2	Known CCS before MI (%)	Known AF before MI	Anticoagulant at Admission	Triggers for Type 2 MI	Outcomes
Arora et al. [39]	T1 MI	775 pts	65 ± ND	ND	ND	ND		In-hospital mortality T1MI vs. T2MI 4.7% vs. 17.4%, *p* < 0.001 30 days mortality after hospital discharge T1MI vs. T2MI 2.2% vs. 11.9%, *p* < 0.001 1 year mortality after hospital discharge T1MI vs. T2MI 12.4% vs. 34.8%, *p* < 0.001
	T2 MI	264 pts	73 ± ND	ND	ND	ND	1. Acute infection: 46% 2. Heart Failure: 11% 3. Anemia: 7% Bradyarrhythmia: ND Multiple provoking conditions: ND	
Guimaraes et al. [40]	T1 MI	11,647 pts	64 ± ND	ND	ND	ND		2-year mortality T1MI vs. T2MI HR (95% IC): 11.82 (5.71–24.46) *p* < 0.0001
	T2 MI	847 pts	66 ± ND	ND	ND	ND	1. AF: 22.4% 2. Anemia: 21.1% 3. Hypotension: 14.5% Bradyarrhythmia: ND Multiple provoking conditions: ND	
Lopez-Cuenca et al. [41]	T1 MI	707 pts	68 ± 13	36% (MI)	15%	ND		1 year mortality T1MI vs. T2MI Adjusted HR (95% IC): 0.88 (0.50–1.53) *p* = 0.692
	T2 MI	117 pts	72 ± 12	50% (MI)	44%	ND	ND	
Javed et al. [42]	T1 MI	143 pts	65.9 ± 1.6	ND	ND	ND		ND
	T2 MI	64 pts	64.2 ± 2.5	ND	ND	ND	1. Sepsis: 20% 2. Heart Failure: 14.5% 3. Tachyarrthythmia: 13.2% Anaemia: 4% Bradyarrhythmia: ND Multiple provoking conditions: ND	
El-Haddad et al. [43]	T1 MI	512 pts	62 (30–97)	ND	ND	ND		In-hospital mortality T1MI vs. T2MI 5.5% vs. 12.5%, *p* < 0.001
	T2 MI	295 pts	64 (22–90)	ND	ND	ND	1. Sepsis: 26% 2. Hypentensive emergency: 15% 3. Gastrointestinal bleeding: 14.5% 4. Tachyarrthythmia: 14.5% Bradyarrhythmia: ND Multiple provoking conditions: ND	
Putot et al. [44]	T1 MI	3710 pts	67 (56–79)	ND	9%	ND		In-hospital mortality T1MI vs. T2MI 5% vs. 11%, *p* < 0.001 In-hospital cardiovascular mortality T1MI vs. T2MI 5% vs. 9%, *p* < 0.001
	T2 MI	862 pts	77 (65–84)	ND	19%	ND	1. Sepsis: 36.5% 2. Tachyarrthythmia: 13% 3. Acute Heart failure: 10% Anaemia: 3% Bradyarrhythmia: 2.5% Multiple provoking conditions: ND	

## References

[1] Kirchhof P., Benussi S., Kotecha D., Ahlsson A., Atar D., Casadei B., Castella M., Christoph-Diener H., Heidbuchel H., Hendriks J. (2016). 2016 ESC Guidelines for the management of atrial fibrillation developed in collaboration with EACTS. Eur. Heart J..

[2] Thygesen K., Alpert J.S., Jaffe A.S., Chaitman B.R., Bax J.J., Morrow D.A., the Executive Group on behalf of the Joint European Society of Cardiology (ESC), American College of Cardiology (ACC), American Heart Association (AHA), World Heart Federation (WHF) Task Force for the Universal Definition of Myocardial Infarction (2018). Fourth Universal Definition of Myocardial Infarction (2018). Circulation.

[3] Januzzi J.L., Sandoval Y. (2017). The Many Faces of Type 2 Myocardial Infarction. J. Am. Coll. Cardiol..

[4] Chiang C.E., Naditch-Brûlé L., Murin J., Goethals M., Inoue H., O’Neill J., Silva-Cardoso J., Zharinov O., Gamra H., Alam S. (2012). Distribution and risk profile of paroxysmal, persistent, and permanent atrial fi-brillation in routine clinical practice insight from the real-life global survey evaluating patients with atrial fibrillation international registry. Circ. Arrhythmia Electrophysiol..

[5] Ruff C.T., Giugliano R.P., Braunwald E., Hoffman E.B., Deenadayalu N., Ezekowitz M.D., Camm A.J., Weitz J.I., Lewis B.S., Parkhomenko A. (2014). Comparison of the efficacy and safety of new oral anticoagulants with warfarin in patients with atrial fibrillation: A meta-analysis of randomised trials. Lancet.

[6] Violi F., Soliman E.Z., Pignatelli P., Pastori D. (2016). Atrial Fibrillation and Myocardial Infarction: A Systematic Review and Appraisal of Pathophysiologic Mechanisms. J. Am. Heart Assoc..

[7] Jabre P., Roger V.L., Murad M.H., Chamberlain A.M., Prokop L., Adnet F., Jouven X. (2011). Mortality associated with atrial fibrillation in patients with myocardial infarction: A systematic review and meta-analysis. Circulation.

[8] He J., Yang Y., Zhang G., Lu X.-H. (2019). Clinical risk factors for new-onset atrial fibrillation in acute myocardial infarction. A systematic review and meta-analysis. Medicine.

[9] Luo J., Li H., Qin X., Liu B., Zhao J., Maihe G., Li Z., Wei Y. (2018). Increased risk of ischemic stroke associated with new-onset atrial fibrillation complicating acute coronary syndrome: A systematic review and meta-analysis. Int. J. Cardiol..

[10] Kundu A., O’Day K., Shaikh A.Y., Lessard D.M., Saczynski J.S., Yarzebski J., Darling C.E., Thabet R., Akhter M.W., Floyd K.C. (2016). Relation of atrial fibrillation in acute myocardial infarction to in-hospital complications and early hospital readmission. Am. J. Cardiol..

[11] Guenancia C., Toucas C., Fauchier L., Stamboul K., Garnier F., Mouhat B., Sagnard A., Lorgis L., Zeller M., Cottin Y. (2018). High rate of recurrence at long-term follow-up after new-onset atrial fibrillation during acute myocardial infarction. Europace.

[12] Lau D.H., Huynh L.T., Chew D.P., Astley C.M., Soman A., Sanders P. (2009). Prognostic impact of types of atrial fibrillation in acute coronary syndromes. Am. J. Cardiol..

[13] Congo K.H., Belo A., Carvalho J., Neves D., Guerreiro R., Pais J.A., Brás D., Carrington M., Piçarra B., Santos A.R. (2019). New-Onset Atrial Fibrillation in St-Segment Elevation Myocardial Infarction: Predictors and Impact on Therapy and Mortality. Arq. Bras. Cardiol..

[14] Jabre P., Jouven X., Adnet F., Thabut G., Bielinski S.J., Weston S.A., Roger V.L. (2011). Atrial fibrillation and death after myocardial infarction: A community study. Circulation.

[15] Hofer F., Kazem N., Hammer A., El-Hamid F., Koller L., Niessner A., Sulzgruber P. (2020). Long-term prognosis of de novo atrial fibrillation during acute myocardial infarction: The impact of anti-thrombotic treatment strategies. Eur. Heart J. Cardiovasc. Pharmacother..

[16] Pokorney S.D., Navar A.M. (2016). In patients with MI, new-onset or existing AF increased risk for CV events at 90 days. Ann. Intern. Med..

[17] Hindricks G., Potpara T., Dagres N., Arbelo E., Bax J.J., Blomström-Lundqvist C., Boriani G., Castella M., Dan G.-A., Dilaveris P.E. (2020). 2020 ESC Guidelines for the diagnosis and management of atrial fibrillation developed in collaboration with the European Association for Cardio-Thoracic Surgery (EACTS): The Task Force for the diagnosis and management of atrial fibrillation of the European Society of Cardiology (ESC) Developed with the special contribution of the European Heart Rhythm Association (EHRA) of the ESC. Eur. Heart J..

[18] Patil S., Gonuguntla K., Rojulpote C., Kumar M., Nadadur S., Nardino R.J., Pickett C. (2020). Prevalence and Determinants of Atrial Fibrillation-associated In-hospital Ischemic Stroke in patients with Acute Myocardial Infarction undergoing Percutaneous Coronary Intervention. Am. J. Cardiol..

[19] Milika A.R., Zorana V.M., Mihailo M.D., Igor M.B., Jovan P.P., Danica M.P., Bosiljka V.T., Sanja S.D., Dragan M.M., Miodrag O.C. (2009). The long-term risk of stroke in patients with acute myocardial infarction complicated with new-onset atrial fibrillation. Clin. Cardiol..

[20] Axelrod M., Gilutz H., Plakht Y., Greenberg D., Novack L. (2020). Early Atrial Fibrillation During Acute Myocardial Infarction May Not Be an Indication for Long-Term Anticoagulation. Angiology.

[21] Stamboul K., Zeller M., Fauchier L., Gudjoncik A., Buffet P., Garnier F., Guenancia C., Lorgis L., Beer J.C., Touzery C. (2014). Incidence and prognostic significance of silent atrial fibrillation in acute myocardial infarction. Int. J. Cardiol..

[22] Wi J., Shin D.-H., Kim J.-S., Kim B.-K., Ko Y.-G., Choi D., Hong M.-K., Jang Y. (2016). Transient New-Onset Atrial Fibrillation Is Associated With Poor Clinical Outcomes in Patients With Acute Myocardial Infarction. Circ. J..

[23] Bang C.N., Gislason G.H., Greve A.M., Bang C.A., Lilja A., Torp-Pedersen C., Andersen P.K., Køber L., Devereux R.B., Wachtell K. (2014). New-Onset Atrial Fibrillation is Associated With Cardiovascular Events Leading to Death in a First Time Myocardial Infarction Population of 89,703 Patients With Long-Term Follow-Up: A Nationwide Study. J. Am. Heart Assoc..

[24] Seet R.C., Friedman P.A., Rabinstein A.A. (2011). Prolonged Rhythm Monitoring for the Detection of Occult Paroxysmal Atrial Fibrillation in Ischemic Stroke of Unknown Cause. Circulation.

[25] Sarkisian L., Saaby L., Poulsen T.S., Gerke O., Hosbond S., Jangaard N., Diederichsen A.C.P., Thygesen K., Mickley H. (2016). Prognostic Impact of Myocardial Injury Related to Various Cardiac and Noncardiac Conditions. Am. J. Med..

[26] Landes U., Bental T., Orvin K., Vaknin-Assa H., Rechavia E., Iakobishvili Z., Lev E., Assali A., Kornowski R. (2016). Type 2 myocardial infarction: A descriptive analysis and comparison with type 1 myocardial infarction. J. Cardiol..

[27] Smilowitz N.R., Subramanyam P., Gianos E., Reynolds H.R., Shah B., Sedlis S.P. (2018). Treatment and outcomes of type 2 myocardial infarction and myocardial injury compared with type 1 myocardial infarction. Coron. Artery Dis..

[28] Sandoval Y., Smith S.W., Sexter A., Thordsen S.E., Bruen C.A., Carlson M.D., Dodd K.W., Driver B.E., Hu Y., Jacoby K. (2017). Type 1 and 2 Myocardial Infarction and Myocardial Injury: Clinical Transition to High-Sensitivity Cardiac Troponin I. Am. J. Med..

[29] Smilowitz N.R., Weiss M.C., Mauricio R., Mahajan A.M., Dugan K.E., Devanabanda A., Pulgarin C., Gianos E., Shah B., Sedlis S.P. (2016). Provoking conditions, management and outcomes of type 2 myocardial infarction and myocardial necrosis. Int. J. Cardiol..

[30] Saaby L., Poulsen T.S., Hosbond S., Larsen T.B., Diederichsen A.C.P., Hallas J., Thygesen K., Mickley H. (2013). Classification of Myocardial Infarction: Frequency and Features of Type 2 Myocardial Infarction. Am. J. Med..

[31] Stein G.Y., Herscovici G., Korenfeld R., Matetzky S., Gottlieb S., Alon D., Gevrielov-Yusim N., Iakobishvili Z., Fuchs S. (2014). Type-II Myocardial Infarction–Patient Characteristics, Management and Outcomes. PLoS ONE.

[32] Baron T., Hambraeus K., Sundström J., Erlinge D., Jernberg T., Lindahl B., TOTAL-AMI study group (2015). Type 2 myocardial infarction in clinical practice. Heart.

[33] Lambrecht S., Sarkisian L., Saaby L., Poulsen T.S., Gerke O., Hosbond S., Diederichsen A.C., Thygesen K., Mickley H. (2018). Different Causes of Death in Patients with Myocardial Infarction Type 1, Type 2, and Myocardial Injury. Am. J. Med..

[34] Cediel G., Gonzalez-Del-Hoyo M., Carrasquer A., Sanchez R., Boqué C., Bardají A. (2017). Outcomes with type 2 myocardial infarction compared with non-ischaemic myocardial injury. Heart.

[35] Shah A.S., McAllister D.A., Mills R., Lee K.K., Churchhouse A.M., Fleming K.M., Layden E., Anand A., Fersia O., Joshi N.V. (2015). Sensitive Troponin Assay and the Classification of Myocardial Infarction. Am. J. Med..

[36] Chapman A.R., Shah A.S.V., Lee K.K., Anand A., Francis O., Adamson P., McAllister D.A., Strachan F.E., Newby D.E., Mills N.L. (2018). Long-Term Outcomes in Patients With Type 2 Myocardial Infarction and Myocardial Injury. Circulation.

[37] Putot A., Derrida S.B., Zeller M., Avondo A., Ray P., Manckoundia P., Cottin Y. (2018). Short-Term Prognosis of Myocardial Injury, Type 1, and Type 2 Myocardial Infarction in the Emergency Unit. Am. J. Med..

[38] Putot A., Jeanmichel M., Chagué F., Avondo A., Ray P., Manckoundia P., Zeller M., Cottin Y. (2019). Type 1 or Type 2 Myocardial Infarction in Patients with a History of Coronary Artery Disease: Data from the Emergency Department. J. Clin. Med..

[39] Arora S., Strassle P.D., Qamar A., Wheeler E.N., Levine A.L., Misenheimer J.A., Cavender M.A., Stouffer G.A., Kaul P. (2018). Impact of Type 2 Myocardial Infarction (MI) on Hospital-Level MI Outcomes: Implications for Quality and Public Reporting. J. Am. Heart Assoc..

[40] Guimarães P.O., Leonardi S., Huang Z., Wallentin L., Van de Werf F., Aylward P.E., Held C., Harrington R.A., Moliterno D.J., Armstrong P.W. (2018). Clinical features and outcomes of patients with type 2 myocardial infarction: Insights from the Thrombin Receptor Antagonist for Clinical Event Reduction in Acute Coronary Syndrome (TRACER) trial. Am. Heart. J..

[41] López-Cuenca Á., Gómez-Molina M., Flores-Blanco P.J., Sánchez-Martínez M., García-Narbon A., Heras-Gómez I.D.L., Sánchez-Galian M.J., Guerrero-Pérez E., Valdés M., Manzano-Fernández S. (2016). Comparison between type-2 and type-1 myocardial infarction: Clinical features, treatment strategies and outcomes. J. Geriatr. Cardiol..

[42] Javed U., Aftab W., Ambrose J.A., Wessel R.J., Mouanoutoua M., Huang G., Barua R.S., Weilert M., Sy F., Thatai D. (2009). Frequency of Elevated Troponin I and Diagnosis of Acute Myocardial Infarction. Am. J. Cardiol..

[43] El-Haddad H., Robinson E., Swett K., Wells G.L. (2012). Prognostic implications of type 2 myocardial infarctions. World J. Cardiovasc. Dis..

[44] Putot A., Jeanmichel M., Chague F., Manckoundia P., Cottin Y., Zeller M. (2020). Type 2 Myocardial Infarction: A Geriatric Population-based Model of Pathogenesis. Aging Dis..

[45] Gupta S., Vaidya S.R., Arora S., Bahekar A., Devarapally S.R. (2017). Type 2 versus type 1 myocardial infarction: A comparison of clinical characteristics and outcomes with a meta-analysis of observational studies. Cardiovasc. Diagn. Ther..

[46] Reid C., Alturki A., Yan A., So D., Ko D., Tanguay J.-F., Bessissow A., Mehta S., Goodman S., Huynh T. (2020). Meta-analysis Comparing Outcomes of Type 2 Myocardial Infarction and Type 1 Myocardial Infarction With a Focus on Dual Antiplatelet Therapy. CJC Open.

[47] Blin P., Fauchier L., Dureau-Pournin C., Sacher F., Dallongeville J., Bernard M.A., Lassalle R., Droz-Perroteau C., Moore N. (2019). Effectiveness and safety of rivaroxaban 15 or 20 mg versus vitamin K antagonists in nonvalvular atrial fibrillation. Stroke.

[48] Raphael C.E., Roger V.L., Sandoval Y., Singh M., Bell M., Lerman A., Rihal C.S., Gersh B.J., Lewis B., Lennon R.J. (2020). Incidence, Trends, and Outcomes of Type 2 Myocardial Infarction in a Community Cohort. Circulation.

[49] Cediel G., Sandoval Y., Sexter A., Carrasquer A., González-Del-Hoyo M., Bonet G., Boqué C., Schulz K., Smith S.W., Bayes-Genis A. (2019). Tarraco Risk Score. A risk estimation in type 2 myocardial infarction and myocardial injury: The Tarraco Risk Score. Am. J. Med..

[50] Shibata T., Kawakami S., Noguchi T., Tanaka T., Asaumi Y., Kanaya T., Nagai T., Nakao K., Fujino M., Nagatsuka K. (2015). Prevalence, clinical features, and prognosis of acute myocardial infarction attributable to coronary artery embolism. Circulation.

